# Reviews and Book Notices

**Published:** 1880-07

**Authors:** 


### Reviews and Book Notices.

ARTICLE IX.—THE TREATMENT OF PUERPERAL SEPTICEMIA BY INTRA-UTERINE INJECTIONS.—By Edward W. Jenks, M.D., LL.D., Chicago, Ill. Reprint from Vol. IV, Gynæcological Transactions, 1880.

This is a paper presented to the American Gynæcological Society by the author, on a very important subject. The reprint before us is a beautifully printed pamphlet of twenty-four pages. The author first gives a full, but concise and interesting history of the practice of introducing remedial substances into the cavity of the uterus for the prevention or cure of septicemia, stating impartially the opinions for and against the practice, with the dangers that have been found to accompany it. He then details the results of his own experience, illustrated by an abstract of three cases in which intra-uterine injections were used with the most marked benefit. His conclusions in reference to the whole subject are clearly and concisely stated in the following propositions which constitute the closing paragraphs of the paper:

1. In its wide-spreading relations to other causes of puerperal diseases, and of death, septicemia stands pre-eminent, for, although puerperal diseases are designated by different names, many lesions of the circulatory, respiratory, and nervous systems are the direct or indirect results of blood poisoning; therefore it is obviously the plain duty of every obstetrician to prevent the absorption of any decomposing materials from the uterus.

2. The objections, which have been made to intra-uterine injections in the treatment of non-puerperal uterine diseases, are not applicable to their use for the prophylaxis or treatment of puerperal septicemia.

3. The number of deaths attributed to intra-uterine injections have, in the majority of instances, occurred when they were used for other purposes than washing out the puerperal uterus with antiseptic fluid.

4. When a death has taken place on account of washing out the uterine cavity after child-birth with a simple antiseptic wash the fatal result has not been in consequence of the injection itself, but from the improper manner of giving it.

5. By the observance of proper precautions on the part of obstetricians this mode of treatment is rendered harmless. To secure entire immunity from danger certain requisites are important, as follows: (*a*) The mouth and neck of the uterus should be well dilated, and a free outlet insured for the injected fluid. (*b*) Air must not be admitted with the injection. (*c*) The fluid should be injected slowly and without much force. (*d*) The fluid used ought not to be of a lower temperature than the normal temperature of the body. (*e*) Powerful astringents should under no circumstances be injected within the uterus, as they are liable to produce contraction of the os and cervix, and thus aid in forcing the injected fluid into the tubes or sinuses.

6. The administration of these injections ought never to be intrusted to a nurse or inexperienced assistant, but should invariably be given by the accoucheur himself, with as much carefulness and attention to every detail as he would in the performance of a surgical operation.

7. Intra-uterine injections should be used invariably succeeding child-birth, if there exist any of the following conditions: (*a*) If there is premature cessation of the lochia with any constitutional disturbance. (*b*) If there exists a purulent or fetid uterine discharge. (*c*) Whenever there is any abnormality of the lochia, or offensive uterine discharge attended by elevation of temperature, or increased frequency of pulse. (*d*) When there are good reasons for believing that the uterus contains fragments of placenta, or is imperfectly contracted, and contains clots or any animal substance.

8, Intra-uterine injections should be more generally used in the prophylaxis and treatment of puerperal diseases, than has heretofore been customary, for the following reasons: (*a*) If properly administered to puerperal women they are devoid of danger and capable of accomplishing results for good which cannot be attained by any other means. (*b*) There are no other modes of treatment or remedial agents which act as speedily in lowering the high temperature of puerperal septicemia, or accomplish better results in certain inflammatory conditions of the uterus peculiar to the puerperal state. (*c*) They are peculiarly serviceable in causing the expulsion of clots, or fragments of placenta, and aid in a marked manner in facilitating the rapid involution of the uterus. (*d*) They have diminished in a remarkable manner the number of deaths, which to all appearance were inevitable from puerperal poisoning—far surpassing in this particular any other known means of treatment.

ARTICLE X.—PAY HOSPITALS AND PAYING WARDS THROUGHOUT THE WORLD. Facts in support of a re-arrangement of the English System of Medical Relief. By Henry C. Burdett, London: J. & A. Churchill. 1879.

Although the author, having a particular object in view, addressed his book specially to the people of England, we have no hesitation to recommend it as very interesting reading to every physician of this country, to every director, trustee or committee of hospital management; in short, to every person who is interested in the proper dispensation of medical charity. The author has evidently devoted much time to his work; he has familiarized himself with the system of hospital management, not only in Great Britain and Ireland, but throughout the world. And, judging by the excellent description of the American systems, we infer he has mastered the details of his great subject with perfect success. The appearance of the book is very opportune at this time, when the medical profession and medical press throughout the land are discussing the great wrongs perpetrated upon our profession by the abuse of medical charity. Those who incline to the belief that the magnitude of these abuses is greatly exaggerated, will find in the statistics drawn from London hospitals the most convincing proofs of an appalling enormity of frauds which may be and are committed if the door of charity is opened indiscriminately to every applicant. Those who try to make the faculties of medical colleges parties of these frauds by acquiescence and encouragement, will probably take their charges into reconsideration after reading this book, which shows conclusively that the abuses reach the same percentage where medical charity is not controlled by medical schools. To all who read the book attentively it must become apparent that these abuses are an inherent feature of all institutions for free medical relief; and that they spring from one common source, to-wit: from that in-born desire of all common people to get everything as cheaply as possible; which desire creates a great aversion to paying for anything they may get free of charge. That London tradesman referred to upon page 153 of the book, presented the whole truth in a nutshell by asserting "that he always gets the best medical advice for his family and himself for a shilling, instead of a guinea. He declared, and indeed boasted, that any one so minded, can get the opinion of the majority of the most eminent consultants in London, with less trouble, and in less time, by paying one shilling to the hospital porter, than by going to their private houses." Change such characters, if you wish to strike at the root of the evil!

The object of the author is "to give facts, and facts only, and to form no conclusion, to urge no scheme of reform which has has not the merit to command success, after a fair trial." And after a careful review of the various systems of hospital administration of this country and Europe; after a fair exposition of their merits and faults, he comes to the conclusion to advocate to his countrymen the adoption of what he is pleased to call "the American plan;" i. e. the system of pay-beds and pay-wards in the general hospitals. "In American hospitals this system is successfully carried out by the resident medical officer or superintendent, who decides, at the time of admission, what a patient should be charged for board. All that he has to guide him in his decision is the evidence produced by the patient, and a scale of payments fixed by the governors. The superintendent cannot fix a lower rate of payments than the lowest amounts on the scale fixed by the trustees. All new cases, on the day after admission, are referred to the visiting committee, who reserve to themselves the right of refusing aid to any person who may apply for relief. This committee regulates the various grades of payment for board, medical attendance, medicine, charges in case of death or removal, and all other expenses incurred by the patient." * * * "If all the large hospitals will give the system a fair trial; if they will each appoint a visiting committee of active, intelligent governors, on the American models, and if they will decide the proportion of free to pay beds at each institution, the thing will be accomplished."

But on one point the author justly insists: "Wherever the pay system is introduced, there must provision be made for the payment, in whole or in part, of the medical officers who attend to the paying patients. No arrangements can be considered satisfactory which do not make provision for the proper payment of the medical staff." But when will this principle be adopted or carried out by the hospital boards? F. C. H.

ARTICLE XI.—THE LAWS OF THERAPEUTICS OR THE SCIENCE AND ART OF MEDICINE. By Joseph Kidd, M.D. "Magna est veritas et prevalebit."

This is a book of two hundred pages, the title of which reads well. One would suppose that at last we had found the one thing desired—something exact—"The Laws of Therapeutics, etc., etc." How sadly we are disappointed the following brief review will indicate:

At page fifty-four ends the first chapter, which is historical. Nothing of importance is introduced and Renouard or Dunglison state all the facts given without the numerous allusions to the "old regime," "exploded theories" and the stereotype inuendoes at the horrors of the "old orthodox distinctive medicine with its rich ornamentations of bleeding, leeching, starving, tartar-emetic and laxatives."

A few new authors are mentioned to bring the history to date, but none with quite the affection, barring a few mistakes which the author corrects, as the "illustrious Hahnemann." Very tenderly does our author refer to the far-seeing eye of genius and the improvements and modifications in the practice of medicine produced by the sharp exclusive teaching of the founder of Homœopathy.

The second chapter on physiology; the third on pathology; and the fourth on the natural history of disease, are brief, and our author rushes forward to the consideration of "Therapeutics and Ars Medica."

The effort is made to demonstrate that Hahnemann was a great and illustrious man in that he discovered the law of *similars*—that he was also a very foolish man, full of mistakes, prejudices and false theories in that he indulged in speculations in regard to *psora* and *infinitessimal doses* of medicine.

Dr. Kidd says at p. 35, et seq.: "Twenty-seven years ago I saw that the essential truth of Hahnemann's law was totally independent of his speculations about dynamization. Adopting with great delight the law of 'similia similibus curantur' as the the chief, though not the only, foundation for therapeutics, I learnt for myself that Hahnemann's 'sober' teaching, the use of the pure, undiluted tinctures, was a far better guide to heal the sick than Hahnemann 'drunk' with mysticism, calling for the exclusive use of infinitesimal doses. The latter I gradually cast aside *in toto* as untrustworthy and unjust to the sick, whose diseases too often remained stationary under treatment by globules but were most effectually and quickly cured by tangible doses of the same medicines which failed to cure when given in infinitesimal doses."

A great part of the book consists in the recital of cases, which are usually prefaced by a history similar to the following: "Capt. S., aged 59, bilious temperament, etc. etc.," given up as hopeless by the *ordinary physicians in the country.* He was with difficulty removed to his mother-in-law's * * * and placed under my care." "Miss ——, aged 26, of a feeble constitution, etc., etc., was attacked with a severe pain across the lumbar region. Anasarca came on in June, with great prostration of strength. Under *ordinary allopathic* treatment she became gradually worse, when she was placed under my care." Guided by this unerring law these patients usually make most remarkable recoveries.

Reiterating the hackneyed expressions in regard to the "partizan spirit of cliques" and the "blunders and bigotry of the old school," the author proceeded to give the latest and most scientific treatment of disease as practiced by the larger number of the most eminent men in that school. He is, in many respects, a *modern* homœopathist.

Many of the cases are interesting and no doubt valuable, but the book is written in bad taste and will hardly add anything to the reputation of the author, whatever that may hitherto have been. The publishers have done their work well. C. W. E.

ARTICLE XII;—ENGLISH HEALTH PRIMERS. American Edition. New York: D. Appleton & Co.

The object of these little books is to disseminate among all classes trustworthy information regarding the conditions and care of health. They are brief, simple and elementary in statement; and each primer is written by a gentleman specially competent to treat his subject. The series deals with hygienic subjects that are of vital importance to everybody, like the following: "Exercise and Training;" "Alcohol: Its Use and Abuse;" "The Home and its Surroundings;" "Premature Death;" "The Skin and its Troubles;" and other topics of equal interest. To bring these little volumes within the reach of everybody who has a few cents to spend and wishes to spend them most profitably, the publishers have put the price at the remarbably low figure of 50 cents per volume.

ARTICLE XIII.—A MANUAL OF AUSCULTATION AND PERCUSSION, EMBRACING THE PHYSICAL DIAGNOSIS OF DISEASES OF THE LUNGS AND HEART AND OF THORACIC ANEURISMS. By Austin Flint, M.D., Professor of the Principles and Practice of Medicine in the Bellevue Hospital Medical College. Second Edition. Henry C. Lea, 1880. Chicago: Jansen, McClurg & Co.

The first edition of this manual was reviewed in these pages a few months ago and what was then said in its commendation is equally applicable to the present volume, which differs very little from the former. The author's name is all that is necessary to recommend this work to the profession of this country.

